# Combined Intrathoracic and Subcutaneous Splenosis Discovered 51 Years after Abdominal Trauma

**DOI:** 10.1155/2015/969067

**Published:** 2015-07-08

**Authors:** James Benjamin Gleason, Anas Hadeh, Maria Julia Diacovo, Jonathan Ryan Schroeder

**Affiliations:** ^1^Department of Pulmonary and Critical Care Medicine, Cleveland Clinic Florida, 2950 Cleveland Clinic Boulevard, Weston, FL 33331, USA; ^2^Department of Pathology and Laboratory Medicine, Cleveland Clinic Florida, 2950 Cleveland Clinic Boulevard, Weston, FL 33331, USA

## Abstract

Splenosis is a rare condition that results from the autotransplantation of splenic parenchyma into unexpected locations such as the abdomen or subcutaneous tissue. In the presence of coexisting injury to the diaphragm intrathoracic transplantation can occur emerging as single or multiple pleural-based masses. This occurs after traumatic rupture of the spleen and is usually asymptomatic, only to be discovered incidentally on routine thoracic or abdominal imaging. To our knowledge this is the third documented case of combined intrathoracic and subcutaneous splenosis found in English literature. This occurred in a 71-year-old male involved in a motor vehicle accident at age 19 requiring urgent splenectomy. He has a significant cigarette smoking history and was referred to our hospital for further evaluation of an abnormality seen on shoulder X-ray.

## 1. Introduction

Splenosis is a rare condition that results from the autotransplantation of splenic parenchyma into unexpected locations such as the abdomen or subcutaneous tissue, usually after traumatic splenic rupture [1]. In presence of coexisting injury to the diaphragm, intrathoracic transplantation can occur as single or multiple pleural-based masses [2]. Pelvic or abdominal splenosis occurs in up to 65% of splenic rupture cases [3] while thoracic implants are significantly less common making up only 18% of splenosis cases [1, 4, 5]. Subcutaneous splenosis is rare with less than 20 reported cases [6].

We report a case of combined intrathoracic and subcutaneous splenosis in a 71-year-old male involved in a motor vehicle accident at age 19 requiring urgent exploratory laparotomy and splenectomy. He had a significant smoking history and was referred to our hospital for further evaluation of an incidental abnormality seen on shoulder X-ray.

## 2. Case Presentation

**Figure 1:**
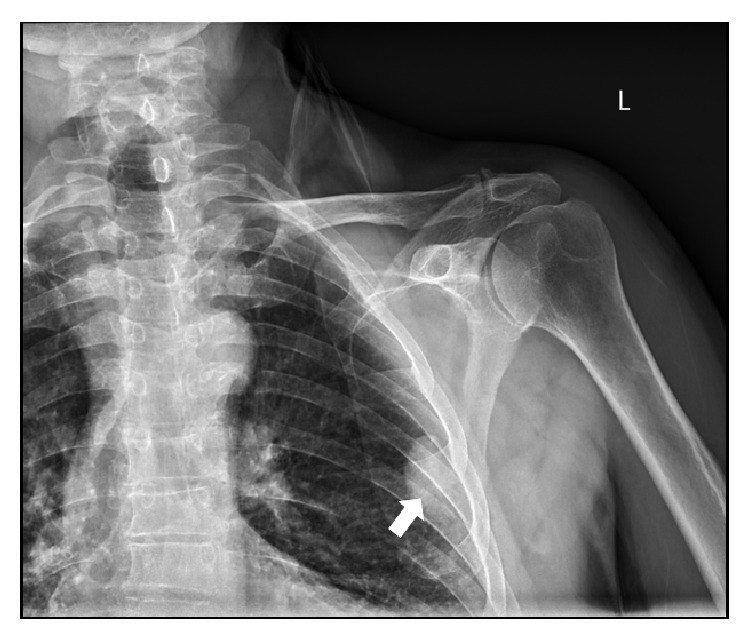
Anteroposterior shoulder X-ray showing left sided pleural-based mass.

**Figure 2:**
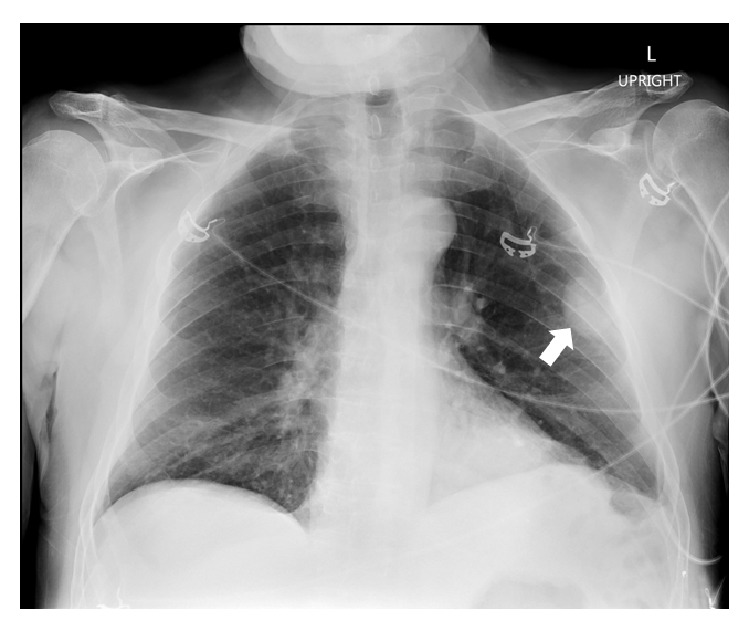
Anteroposterior Chest X-ray showing left sided pleural-based mass.

A 71-year-old Hispanic male was referred to our institution for the evaluation of an incidental 4 cm left peripheral mid-lung opacity on shoulder X-ray (Figure 1) as well as followup chest X-ray (Figure 2). His past medical history was remarkable hypertension. Prior surgeries included an urgent exploratory laparotomy and splenectomy due to motor vehicle collision at age 19. He is a former smoker with a 40-pack-year history and quit 10 years before presentation. He disclosed no significant occupational or environmental exposures.

**Figure 3:**
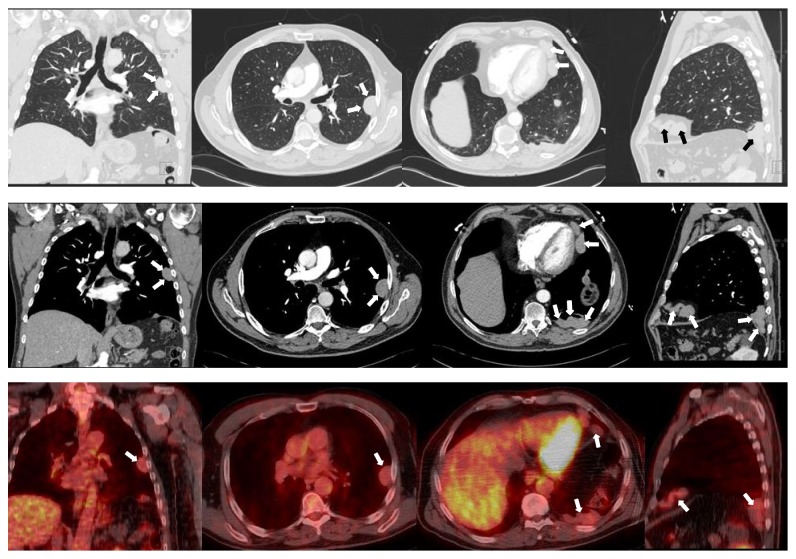
Top row: CT Chest with IV contrast, pulmonary windows. Middle row: CT Chest with IV contrast, soft tissue windows. Bottom row: fused PET/CT showing FDG uptake in the corresponding lesions. Columns (from left to right): coronal views highlighting the peripheral left mid-lung pleural-based lesion. Axial views highlighting the same peripheral left mid-lung pleural-based lesions. Axial views highlighting the pleural-based lesions at the left heart border and the posterior aspect of the left lower lobe. Sagittal views demonstrating pleural-based lesions at the anterior and posterior regions of the left hemidiaphragm.

**Figure 4:**
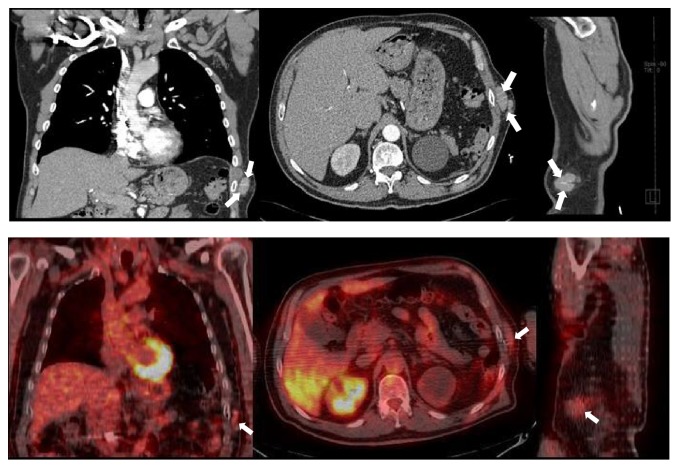
Top row (from left to right): coronal, axial, and sagittal CT Chest with IV contrast soft tissue views highlighting the left chest wall subcutaneous lesions. Bottom row (from left to right): coronal, axial, and sagittal fused PET/CT showing FDG uptake to the same lesions.

At the time of evaluation, he did not have any respiratory symptoms. His physical examination was unremarkable with the expected healed surgical scar at the left lateral abdomen. A computed tomography (CT) scan of the chest was ordered to better characterize the incidental lesion. This study revealed multiple pleural-based noncalcified masses, the largest measuring up to 7.2 centimeters, mild centrilobular and paraseptal emphysema, and two subcutaneous masses at the site of his 51-year-old healed abdominal incision (Figures 3 and 4). Thoracic and subcutaneous splenosis were suspected due to his history of abdominal trauma and the nature of the lesions on CT imaging but there was substantial concern for malignant tumor or pleural metastasis. We proceeded with CT-guided core needle lung biopsy of the most accessible thoracic lesions located in the posterior aspect of the left lower lobe of the lung. The needle biopsy revealed histiocytes and nonspecific chronic inflammation and was considered to be nondiagnostic.

F-fluoro-2-deoxygluocose (FDG) positron emission tomography/CT (PET/CT) revealed the previously detected pleural masses to be only mildly FDG-avid (Figure 3). There were no abnormally active mediastinal or hilar lymph nodes. A similarly FDG-avid focus along the L rib cage was also reported at the site of his healed abdominal incision (Figure 4).

**Figure 5:**
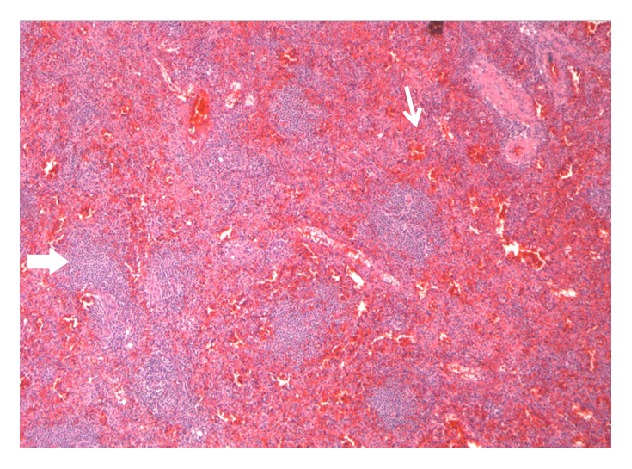
Hematoxylin & Eosin stain at 5x magnification. Splenic parenchyma composed of red and white pulp. Thick arrow: white pulp with primary and secondary follicles composed of mature B-lymphocytes. Thin arrow: red pulp with sinuses, vasculature, and cords of Billroth.

**Figure 6:**
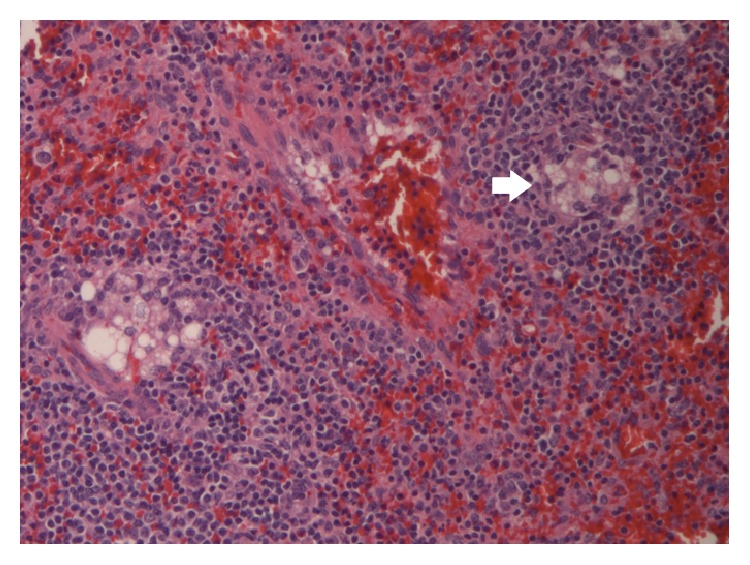
Hematoxylin & Eosin stain at 20x magnification identifying lipogranulomas present in our case.

**Figure 7:**
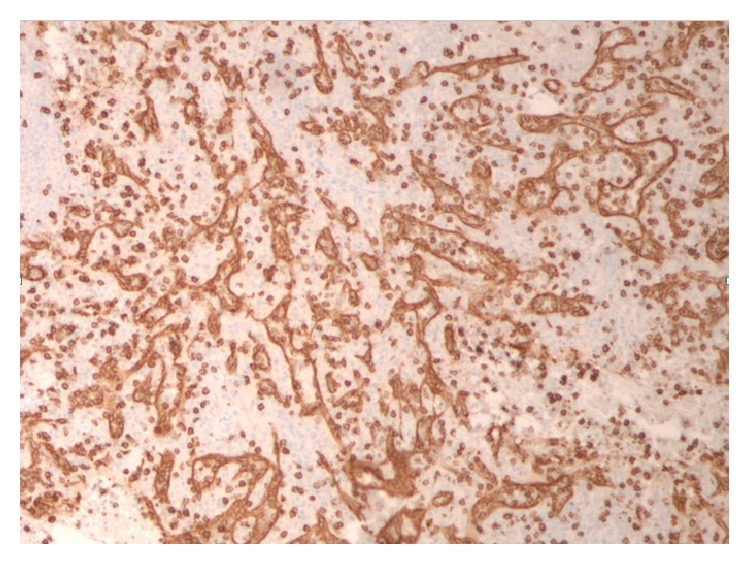
CD8 immunostain of red pulp. Highlights endothelial cells lining the splenic sinuses (littoral cells) and rare cytotoxic T-cells. The spaces between the sinuses are the splenic cords (of Billroth).

Because of his risk factors for primary lung malignancy he underwent left sided video-assisted thoracoscopic surgery (VATS) and biopsy. Surgical specimens demonstrated pleural-based masses which were grossly friable and maroon-tanned. Histologically, two well-delineated areas compatible with white and red pulp were noted (Figure 5). Occasional lipogranulomas were present on the red pulp (Figure 6). Immunophenotyping by immunohistochemistry and flow cytometry analysis revealed a preserved immunoarchitecture and absence of an abnormal B- or T-cell population, respectively (Figure 7). These findings supported the diagnosis of thoracic splenosis.

## 3. Discussion

Splenosis is a unique, acquired condition first described by von Kuttner in 1910 [7]. It results from the autotransplantation of splenic parenchyma into unexpected locations such as the abdomen, pleural space, or subcutaneous tissues. The exact pathogenesis of splenosis is unknown but is suggested to be related to mechanical trauma and splenic rupture releasing splenic pulp into the surrounding tissues [1]. Another proposed mechanism is hematogenous spread as has been postulated in reported cases of intrahepatic [8] and cerebral splenosis [9]. For this reason, splenosis can occur anywhere in the body but is most frequently found in the intraperitoneal space and may be present in as many as 65% of splenic rupture cases [3].

In the presence of coexisting diaphragm abnormalities or injury intrathoracic implantation may occur leading to one or more pleural-based splenic nodules [2]. This was first described by Shaw and Shafi as an autopsy finding in 1937 [10]. Thoracic splenosis is much less frequent and has been described less than 60 times in English literature although; it is estimated to occur in up to 18% of patients following traumatic splenic rupture [4]. In 75% of these cases there are multiple pleural-based splenic nodules [4]. Due to the location of the spleen and anatomic boundaries intrathoracic autotransplantation almost always occurs into the left hemithorax. The overwhelming majority of thoracic splenosis cases occur in males and this phenomenon is probably related to the higher incidence of risky behavior and trauma in men [4]. In addition to subpleural ectopic implants there have also been pulmonary intraparenchymal implants in patients with concurrent pulmonary laceration [11, 12].

Subcutaneous splenosis is exceedingly rare with less than 20 reported cases, 16 of which were recently reviewed by Papakonstantinou et al. They found that the majority of cases occur after gunshot wounds or, as with our patient, occur at the site of abdominal surgical scars [6].

Splenosis is generally an asymptomatic condition but there have been thoracic cases presenting with pleurisy [11] and hemoptysis [12]. Intra-abdominal cases have presented with bowel obstruction [13], GI hemorrhage [14, 15], and compression of other neighboring organs [16]. For this reason, splenosis is usually discovered incidentally after general screening or during the workup of another problem such as our case.

In patients with history highly suggestive of splenosis and without risk factors or symptoms indicative of malignancy diagnosis can be obtained nonsurgically using radionucleotide scintigraphy or Magnetic Resonance Imaging (MRI). ^99m^Technetium (Tc) labeled heat-damaged erythrocyte scanning is the preferred method because it is significantly more specific than ^99m^Technetium sulfur colloid, indium 111-labeled platelet, or ^99m^white blood cell scanning due to diminished liver uptake [4, 17, 18].

An alternative noninvasive technique is Ferumoxide MRI [19]. This method uses superparamagnetic iron oxide as a marker by virtue of its accumulation in reticuloendothelial tissue for processing [20]. Ferumoxide MRI is employed less frequently and no studies have been published which directly compare its diagnostic sensitivity and specificity to the more commonly used ^99m^heat-damaged erythrocyte radionucleotide scanning. In general, MRI has significantly better resolution when compared to other imaging modalities and for this reason may be superior in the diagnosis of splenosis [1].

In cases similar to our own where there is suspicion, malignancy risk factors, or inconclusive imaging studies a tissue biopsy should be obtained for definitive diagnosis. CT-guided fine needle Aspiration (FNA) could be employed when nodules are anatomically amenable. Unfortunately, without the histologic architecture provided by a resection, a small needle biopsy or FNA would not be able to differentiate the splenic elements from chronic inflammation, an intraparenchymal lymph node or a low grade lymphoma. However, the most important contributory information would be the absence of a carcinoma, mesothelioma, or metastatic disease. Ultimately, for thoracic lesions video-assisted thoracoscopic surgery (VATS) with biopsy can be used to obtain a tissue diagnosis. In very rare symptomatic cases this approach is diagnostic as well as therapeutic. Fine needle aspiration or surgical biopsies allow cytopathological analysis which may potentially rule out primary pulmonary malignancies, pleural malignancies, pleural-based metastatic disease, lymphomas, and other varieties of cancer. Histologically ectopic splenic tissue is similar to normal spleen with encapsulated parenchyma consisting of red and white pulp with lymphoid follicles.

Because splenosis is generally asymptomatic, surgical excision of ectopic splenic nodules is often unnecessary and may subject patients to unnecessary surgical risks [21–23]. Moreover, it is well-known that complete splenectomy is associated with higher rates of infection by encapsulated organisms such as* Neisseria meningitidis*,* Streptococcus pneumoniae*, and* Haemophilus influenzae* [24]. Despite the paucity of usual splenic anatomy in patients with splenosis, the existence of ectopic splenic tissue is hypothesized to provide some level of immunity above splenectomized patients, opposing advocates of surgical excision. This hypothesis has been strengthened by research demonstrating a greater than twofold increase in IgM and IgG antibody levels leading to augmented opsonization and serum pathogen clearance in patients with splenosis compared to splenectomized only patients [25]. Anecdotally, peripheral blood smears in patients with splenosis may also lack Howell-Jolly bodies suggesting normal immune function [26]. Nevertheless, the immunologic functionality of ectopic splenic tissue in splenosis is still being debated. More recently, animal research has presented evidence that ectopic splenic tissue is incompetent and may contribute to higher mortality in sepsis [27]. Considering that neither the amount of ectopic splenic tissue necessary nor the level of immunity this tissue endows is known [28] it is still recommended to administer penicillin prophylaxis and vaccinations in patients with splenosis [24].

## 4. Conclusion

To conclude, we present the third documented case of combined thoracic and subcutaneous splenosis [29, 30]. This is a relatively benign condition that should be strongly considered in patients with a history of abdominal trauma who present for incidental masses or nodules on X-Ray or CT imaging. Typically, these masses will be in the abdomen but if diaphragmatic trauma has occurred it is possible left sided pleural-based nodules may be present. Splenosis can be noninvasively diagnosed with ^99m^Tc labeled heat-damaged erythrocyte scintigraphy or Ferumoxide MRI. Should risk factors for malignancy be present or the aforementioned studies be inconclusive a CT-guided FNA or VATS with biopsy can be performed to rule out malignancy and obtain a definitive diagnosis. Even though the immunologic functionality of ectopic splenic tissue is debated surgical excision is not recommended unless the patient is symptomatic.
